# Clinical Characteristics of Patients With IgG4-Related Disease Complicated by Hypocomplementemia

**DOI:** 10.3389/fimmu.2022.828122

**Published:** 2022-02-24

**Authors:** Yuya Fujita, Shoichi Fukui, Masataka Umeda, Sosuke Tsuji, Naoki Iwamoto, Yoshikazu Nakashima, Yoshiro Horai, Takahisa Suzuki, Akitomo Okada, Toshiyuki Aramaki, Yukitaka Ueki, Akinari Mizokami, Tomoki Origuchi, Hiroshi Watanabe, Kiyoshi Migita, Atsushi Kawakami

**Affiliations:** ^1^ Department of Immunology and Rheumatology, Division of Advanced Preventive Medical Sciences, Nagasaki University Graduate School of Biomedical Sciences, Nagasaki, Japan; ^2^ Department of Rheumatology, Fukushima Medical University School of Medicine, Fukushima, Japan; ^3^ Department of Internal Medicine, Sasebo City General Hospital, Nagasaki, Japan; ^4^ Department of General Internal Medicine and Rheumatology, Clinical Research Center, National Hospital Organization (NHO) Nagasaki Medical Center, Nagasaki, Japan; ^5^ Department of Rheumatology, Japanese Red Cross Nagasaki Genbaku Hospital, Nagasaki, Japan; ^6^ Rheumatic Disease Center, Sasebo Chuo Hospital, Nagasaki, Japan; ^7^ Department of Rheumatology, Japan Community Healthcare Organization, Isahaya General Hospital, Nagasaki, Japan

**Keywords:** complement C3, complement C4, IgG, IgG4, IgG4-RD

## Abstract

**Background:**

A proportion of patients with immunogloblin G (IgG) 4-related disease (IgG4-RD) have hypocomplementemia. We aimed to identify characteristics of such patients.

**Methods:**

We analyzed the demographic and clinical data and complement levels of 85 patients with IgG4-RD. We defined hypocomplementemia as serum C3 and/or C4 levels below the lower limit of normal at diagnosis. We also compared the characteristics of patients with and without IgG4-RD.

**Results:**

Thirty-two (38%) patients had hypocomplementemia at diagnosis. Patients with hypocomplementemia had more lymph node (p < 0.01), lung (p < 0.01), and kidney (p = 0.02) involvement and a higher IgG4-RD responder index than those without (p = 0.05). Additionally, patients with hypocomplementemia had significantly higher IgG (p < 0.01), IgG4 (p < 0.01), and soluble interleukin 2-receptor (sIL-2R) (p < 0.01) levels and total IgG minus IgG4 (p < 0.01). C3 and C4 levels negatively correlated with IgG, IgG4, and sIL-2R levels, total IgG minus IgG4, and number of IgG4-RD responder index: a measure of the disease activity in IgG4-RD. Patients with hypocomplementemia at diagnosis had a significantly higher frequency of relapse (p = 0.024), as determined using the log-rank test. A multivariate logistic regression analysis showed the presence of hypocomplementemia was independently associated with relapse (OR, 6.842; 95% confidence interval [95%CI], 1.684–27.79; p = 0.007).

**Conclusions:**

Patients with IgG4-RD with hypocomplementemia have a more active clinical phenotype, suggesting contributions of the complement system in the pathophysiology of IgG4-RD.

## Introduction

Immunogloblin G (IgG) 4-related disease (IgG4-RD) is a systemic condition characterized by elevated serum IgG4. Involved tissues exhibit IgG4-positive lymphoplasmacytic infiltrates and fibrosis on histopathology (1). IgG is subdivided into four subtypes: IgG1, IgG2, IgG3 and IgG4. Although serum IgG4 levels account for 1%-7% in IgG subclasses, the proportion of IgG4 in IgG subclasses is significantly increased in patients with IgG4-RD (2). The pathogenicity of IgG4 is still unclear in IgG4-RD. Not IgG4 but IgG1 is reported to initiate destructive change to affected organs (2). Organs affected with IgG4-RD include the pancreas, lacrimal glands, salivary glands, kidneys, lungs, retroperitoneum, aorta, skin, and lymph nodes. Although the pathogenesis of IgG4-RD is poorly understood, investigations of a wide variety of cellular and humoral abnormalities, including immune cells such as T cells, B cells, IgG4, plasmablasts, and M2 macrophages, are providing new insight (2). The infiltrating M2 macrophages to multiple lesions are associated with the production of pro-fibrotic cytokines [interleukin-10 (IL-10), IL-33] and chemokines [cc-chemokine ligand 18 (CCL18)] *via* type 2 helper T lymphocytes (2). Complement is a factor that potentially contributes to IgG4-RD pathogenesis (3).

Hypocomplementemia, defined as having C3 or C4 complement levels below the lower limits of normal, is one of the serological features of IgG4-RD. More than one-third of Japanese patients with IgG4-RD have hypocomplementemia (4), as do one-fourth of patients with active IgG4-RD in the United States (5). In a small retrospective study in Japan, 3 of 14 patients with IgG4-related kidney disease showed decreased complement levels on disease relapse (6). However, few reports have been published on the clinical features and prognosis of IgG4-RD patients with or without hypocomplementemia at diagnosis. Hence, we performed this study to compare the clinical features of patients with IgG4-RD with and without concomitant hypocomplementemia.

## Materials and Methods

### Study Subjects

A total of 107 patients diagnosed with IgG4-RD between December 2008 and September 2021 were recruited from the Department of Immunology and Rheumatology of Nagasaki University Hospital, the Department of Rheumatology of Fukushima Medical University Hospital, and affiliated hospitals. Twenty-two IgG4-RD patients whose complement levels were not measured at diagnosis were excluded. All patients fulfilled the 2020 revised comprehensive diagnostic criteria for IgG4-RD (7). Finally, 85 consecutive patients with IgG4-RD were included in the study.

### Data and Image Preparation

We collected patients’ demographic and clinical data from their medical records and laboratory findings at diagnosis. All patients were examined by rheumatologists certified by the Japan College of Rheumatology. Clinical relapse was determined from their medical records. We defined hypocomplementemia as serum C3 levels and/or C4 levels below the lower limit of normal at our hospitals (i.e., C3 < 73 mg/dL, C4 < 11 mg/dL). Disease activity was determined based on the IgG4-RD responder index (IgG4-RD RI) (8). We obtained computed tomography, positron emission tomography, and magnetic resonance imaging scans, and gallium scintigraphy data to assess lacrimal gland, salivary gland, lymph node, thyroid, lung, pancreas, retroperitoneum, aorta, and kidney involvement, in addition to reviewing physical examination results. Only two patients had no imaging data available. Clinical relapse was defined as [1] a recurrence of symptoms and signs and/or worsening of imaging studies, with or without re-elevation of the serum IgG4 level and [2] an increase of the prednisolone dose or [3] addition of an immunosuppressant (9).

### Statistical Analysis

Data were analyzed with SPSS Statistics software version 22.0 (IBM Corp., Armonk, NY). Results were nonnormally distributed and compared with the Mann–Whitney U test and are presented throughout the manuscript with median and 25^th^–75^th^ centiles (median, interquartile range [IQR]). Spearman’s correlation coefficient was used to evaluate correlations. The chi-square test was used to compare categorical variables represented as frequencies. The relapse-free survival analysis was assessed using Kaplan–Meier analysis, with the significance based on the log-rank test.

We compared the characteristics of patients with IgG4-RD with hypocomplementemia (n = 32) to those without (n = 53). Categorical variables are described as frequencies, whereas quantitative variables are described as medians and IQR. Associations between the variables were assessed using Fisher’s exact test for categorical variables and Wilcoxon’s rank-sum test for quantitative variables. Relationships between variables were analyzed using Spearman’s rank correlation. The prognostic factors for relapse were identified using a stepwise multiple logistic regression model. All tests were two-sided, and p-values of <0.05 were considered significant.

The study was approved by the Institutional Review Board of Nagasaki University Hospital (17091109), Fukushima Medical University (29317), and affiliated hospitals. Informed consent for data use was obtained from some of the patients, and an opt-out procedure was used for the rest.

## Results

### Clinical Characteristics of Patients With IgG4-RD

The demographic and clinical characteristics of all 85 patients with and without hypocomplementemia at diagnosis are summarized in **Table 1**.

**Table 1:** Demographic, clinical, and laboratory characteristics of patients with immunoglobulin 4-related disease diagnosed with or without hypocomplementemia.

	All patients (n = 85)	With hypocomplementemia (n = 32)	Without hypocomplementemia (n = 53)	p-value
Characteristics				
Age, median (IQR)	66 (59 – 72)	66 (59 – 74)	65 (58 – 72)	0.59
Male sex, n (%)	62 (73)	25 (78)	37 (70)	0.4
BMI, median (IQR)	22 (20 – 25)	22 (20 – 25)	22 (20 – 26)	0.73
Allergy, n (%)	23 (27)	6 (19)	17 (33)	0.16
Laboratory test, median (IQR)				
White blood cells, ×10^3^/μL	6.0 (4.8 – 7.4)	5.7 (4.4 – 6.8)	6.7 (5.1 – 7.4)	0.07
Eosinophils, ×10^3^/μL	230 (111 – 370)	243 (116 – 478)	204 (109 – 360)	0.39
Hemoglobin, g/dL	13.1 (12.2 – 14.4)	12.8 (11.6 – 14.1)	13.1 (12.7 – 14.5)	0.14
Platelets, ×10^4^/μL	22.4 (18.5 – 25.5)	20.8 (17.4 – 24.2)	23.3 (19 – 26)	0.09
C-reactive protein, mg/dL	0.21 (0.07 – 0.34)	0.28 (0.14 – 0.32)	0.12 (0.05 – 0.36)	0.17
Creatinine, mg/dL	0.8 (0.68 – 0.95)	0.83 (0.70 – 1.19)	0.8 (0.67 – 0.91)	0.17
eGFR (mL/min/1.73 m^2^)	69 (57 – 81)	59 (42 – 81)	70 (61 – 81)	0.09
IgG, mg/dL	2055 (1585 – 2862)	2717 (2227 – 4130)	1738 (1461 – 2227)	<0.01
IgG4, mg/dL	480 (212 – 945)	773 (410 – 1713)	385 (186 – 687)	<0.01
Total IgG minus IgG4, mg/dL	1522 (1173 – 2106)	2063(1639 – 2583)	1307 (1068 – 1588)	<0.01
IgE, IU/mL	244 (93 – 759)	239 (84 – 659)	257 (111 – 1110)	0.51
Soluble IL-2 receptor, U/mL	758 (473 – 1251)	1145 (1000 – 1699)	584 (391 – 828)	<0.01
Involved organs, n (%)				
Orbit and lacrimal gland	22 (27)	7 (23)	15 (29)	0.06
Salivary gland	48 (58)	19 (59)	29 (56)	0.74
Lymph node	44 (52)	23 (72)	21 (40)	<0.01
Thyroid	4 (5)	2 (6)	2 (4)	0.601
Lung	12 (14)	9 (28)	3 (6)	<0.01
Pancreas	16 (17)	7 (22)	9 (17)	0.58
Bile duct and liver	2 (2)	0 (0)	2 (4)	0.27
Kidney	16 (19)	10 (31)	6 (11)	0.02
Aorta and large blood vessel	10 (12)	3 (9)	7 (13)	0.6
Prostate	6 (8)	3 (10)	3 (6)	0.53
Retroperitoneum	23 (27)	8 (25)	15 (28)	0.74
Numbers of involved organs, median (IQR)	2 (1 – 3)	2 (2 – 4)	2 (1 – 3)	0.07
IgG4-RD responder index	9 (6 – 12)	9 (9-15)	9 (6 – 12)	0.05

BMI, body mass index; GFR, glomerular filtration rate; IgG, immunoglobulin G; IL-2, interleukin-2; IgG4-RD, immunoglobulin G4-related disease; IQR, interquartile range.

Thirty-two patients (38%) were classified in the hypocomplementemia group. In the hypocomplementemia group, 22 patients (69%) had C3 and C4 reduction, 3 (9%) had only C3 reduction, and 7 (22%) had only C4 reduction.

The median age at diagnosis was 66 years; 62 patients were men (73%). There were no significant differences in age at diagnosis, sex, body mass index, and history of allergy between the two groups.

**Figure 1:**
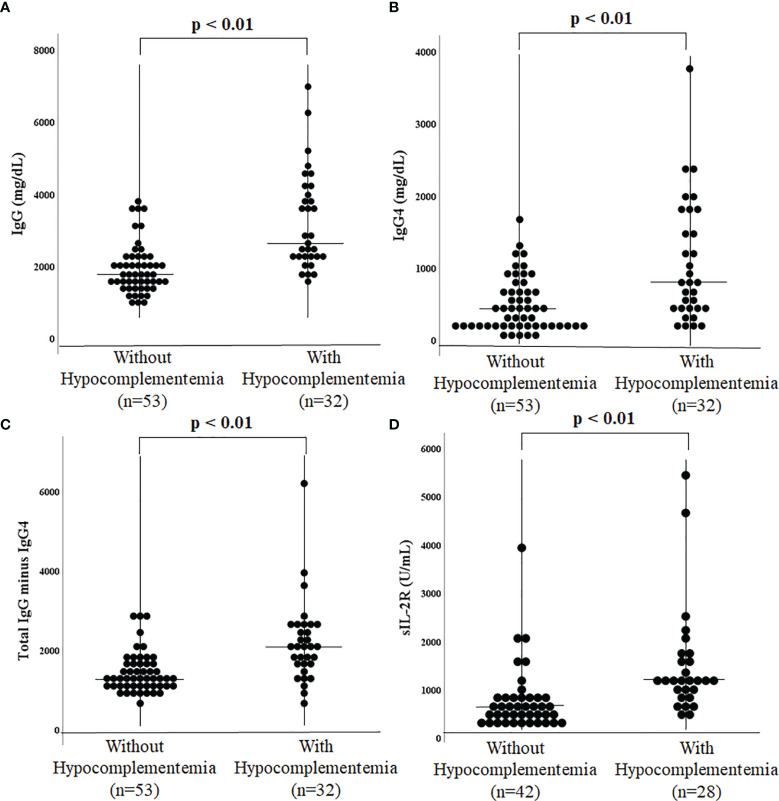
Comparison of immunoglobulin G4-related disease with and without hypocomplementemia. Serum IgG **(A)**, IgG4 **(B)**, total IgG minus IgG4 **(C)**, and soluble IL-2R **(D)** in patients with hypocomplementemia were significantly higher than those in patients without hypocomplementemia.

The hypocomplementemia group had significantly higher IgG (2717 [2227–4130] vs. 1738 [1461–2227] mg/dL, p < 0.01) (**Figure 1A**) and IgG4 levels (773 [410–1713] vs. 385 [186–687] mg/dL, p < 0.01) (**Figure 1B**). Total IgG minus the IgG4 value was also significantly higher in the hypocomplementemia group (2063 [1639–2539] vs. 1307 [1068–1588] mg/dL, p < 0.01) (**Figure 1C**). Additionally, patients with hypocomplementemia had significantly higher soluble IL-2 receptor (sIL-2R) levels (1145 [1000–1699] vs. 584 [391–828] U/mL, p < 0.01) (**Figure 1D**). There were no differences in complete blood count, including the absolute number of eosinophils and renal function markers. Finally, a comparison of the initial prednisolone dose showed no significant difference between the hypocomplementemia and normal complement groups (30 [20–35] vs. 30 [28–35] U/mL, p = 0.154).

### Comparison of Affected Organs Between Patients With and Without Hypocomplementemia

The hypocomplementemia group had significantly more lymph node (23 [72%] vs. 21 [40%], p < 0.01), lung (9 [28%] vs. 3 [6%], p = 0.01), and kidney (10 [31%] vs. 6 [11%], p < 0.02) involvement. Patients with hypocomplementemia also had a significantly higher IgG4-RD responder index (9 [9–15] vs. 9 [6–12], p = 0.05) (**Table 1**).

### Correlations Between C3, C4, and Clinical Characteristics at Baseline

**Figure 2:**
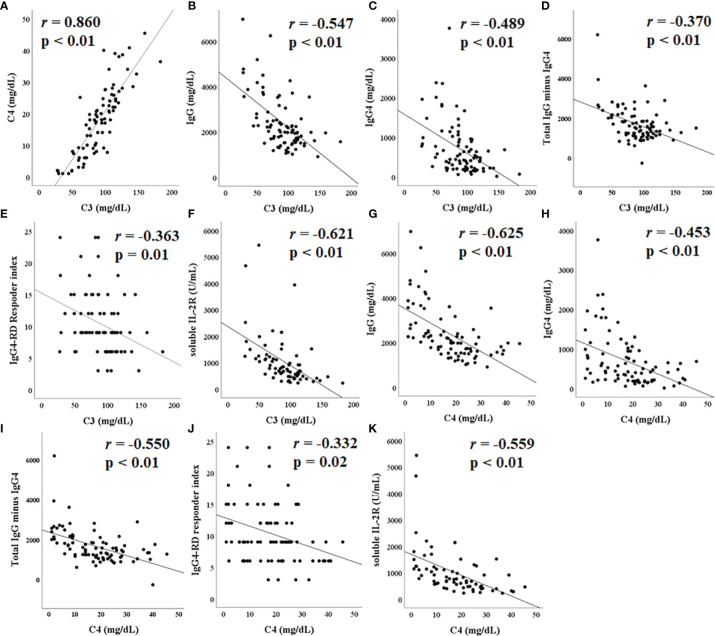
Correlation between complement C3/C4 and other laboratory tests. C3 levels positively correlated with C4 **(A)**. C3 levels negatively correlated with immunoglobulin **(IgG) (B)**, IgG4 **(C)**, total IgG minus IgG4 **(D)**, immunoglobulin G4-related disease (IgG4-RD) responder index **(E)**, and soluble IL-2R **(F)**, all significantly. Similarly, C3 levels showed a significant negative correlation with IgG **(G)**, IgG4 **(H)**, total IgG minus IgG4 **(I)**, IgG4-RD responder index **(J)**, and soluble IL-2R **(K)**.

The correlations between C3 levels, C4 levels, and other biological measures are presented in **Figure 2**. C3 levels positively correlated with C4 levels (r = 0.860, p < 0.01) (**Figure 2A**). C3 levels inversely correlated with IgG levels (r = −0.547, p < 0.01), IgG4 levels (r = −0.489, p < 0.01), total IgG minus IgG4 value (r = −0.370, p < 0.01), the number of IgG4-RD RI (r = −0.363, p = 0.01), and the level of soluble IL-2 receptor (r = −0.621, p < 0.01); these inverse correlations were significant (**Figures 2B–F**). Similarly, C4 levels inversely correlated with IgG levels (r = −0.625, p < 0.01), IgG4 levels (r = −0.453, p < 0.01), total IgG minus IgG4 value (r = −0.550, p < 0.01), the number of IgG4-RD RI (r = −0.332, p = 0.02), and the level of soluble IL-2 receptor (r = −0.559, p < 0.01) (**Figures 2G–K**). There was no significant correlation between C3 and C4 levels and the estimated glomerular filtration rate.

### Comparison of Relapse in Patients With IgG4-RD

**Figure 3:**
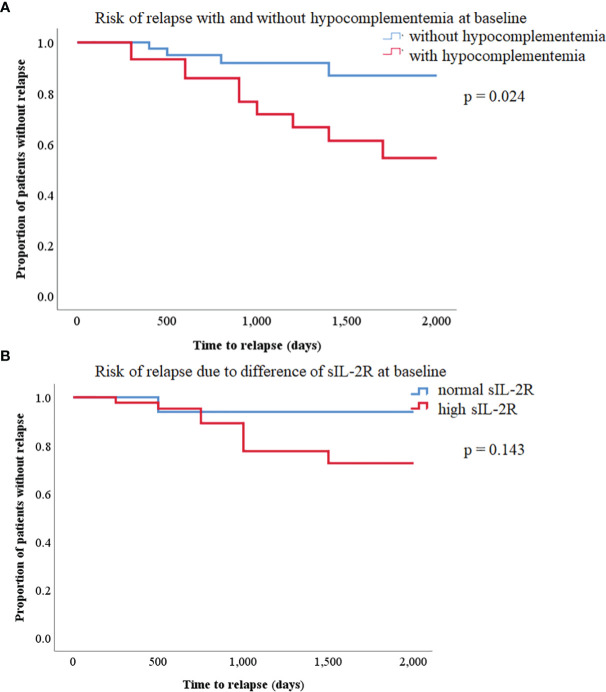
**(A)** Kaplan–Meier curves of relapse-free survival in patients with immunoglobulin G4-related disease (IgG4-RD) with or without hypocomplementemia. There was a statistically significant difference between the two curves (log-rank test, p = 0.024). **(B)** Kaplan–Meier curves of relapse-free survival between high and normal soluble IL-2R in patients with IgG4-RD. There was no significant difference between the two curves (log-rank test, p = 0.143).

Kaplan–Meier survival analysis on disease relapse is shown in **Figure 3A**. The log-rank test showed that patients with hypocomplementemia had a significantly higher relapse risk of IgG4-RD than those without hypocomplementemia (p = 0.024). Similarly, patients with IgG4-RD with elevated serum sIL-2R levels tend to relapse (**Figure 3B**). We attempted to identify the clinical parameters associated with relapse by performing a multivariate logistic regression analysis (**Table 2**). The presence of hypocomplementemia was independently associated with relapse (OR, 6.842; 95% confidence interval [95%CI], 1.684–27.79; p = 0.007).

**Table 2:** A multivariable logistic analysis between with and without relapse.

	With relapse (n = 16)	Without relapse (n = 69)	P*	P**	OR (95% CI)
Hypocomplementemia (%)	11 (68.8)	21 (30.4)	0.008	0.007	6.842 (1.684 - 27.79)
sIL-2R median(IQR)	1110 (701 - 1929)	692(454 - 1142)	0.045	–	–
IgG4-responder index median(IQR)	12 (9 - 12)	9 (6 - 12)	0.271	–	–

95%CI, 95% confidence interval; OR, odds ratio.

*Bivariable analysis (Fisher’s exact test for categorical variables

and Wilcoxon’s rank-sum test for quantitative variables).

**Multivariable analysis (stepwise logistic regression model).

## Discussion

Hypocomplementemia was previously reported to occur concomitantly with IgG4-RD (4, 10, 11). This study revealed that relapse could be predicted in advance using complement C3/C4 at baseline. Peng et al. reported no differences in relapse-free survival between patients with and without hypocomplementemia (11), whereas our study revealed that patients with hypocomplementemia had higher subsequent relapse-free survival of IgG4-RD episodes. Although the number of cases was limited, the presence of hypocomplementemia was associated with relapse in a multivariate logistic regression analysis. The initial dose of prednisolone in the cohort of Peng et al. was significantly higher in patients with hypocomplementemia than in those without hypocomplementemia (11). Our cohort had no differences in the initial prednisolone dose between the two groups. This difference might influence the relapse rate in the two studies. From this point of view, IgG4-RD patients with hypocomplementemia tend to have a serious disease course whereas IgG4-RD patients with normal complement levels may be mild disease course with less relapse rates.

The mechanism of hypocomplementemia in IgG4-RD remains unclear. Human IgG is subdivided into four classes, which differ markedly in activating the classic complement pathway (12). IgG1 and IgG3 can activate complement effectively; IgG2 only activates complement when the target antigen concentrations are high (12, 13). However, IgG4 cannot activate complement (12, 13). Muraki et al. reported that circulated immunocomplex (CIC) determined by C1q assay was significantly associated with serum IgG1 levels in autoimmune pancreatitis (14). Serum CIC levels and serum IgG1 levels in autoimmune pancreatitis are significantly higher than in healthy controls (14). Furthermore, elevated serum CIC and IgG1 concentrations are associated with decreased C3 and C4 levels (14). This finding suggests that although IgG4 may have contributed little to complement activation, IgG1 played a prominent role by forming an IgG1-type immune complex *via* the classical pathway (14).

Similarly, hypocomplementemia in IgG4-related kidney disease is related to the elevation of IgG subclasses other than IgG4, including IgG1 (15). These studies suggest that not IgG4 but other types of IgG result in hypocomplementemia. In our study, the significant association between the value of total IgG minus IgG4 and complement support the fact that IgG subclasses other than IgG4 can be responsible for hypocomplementemia in IgG4-RD. In contrast, Sugimoto et al. reported as follows; [1] Immunocomplex (IC) in sera isolated from IgG4-RD patients contains IgG4 and IgM, [2] IC isolated from IgG4-RD patients with hypocomplementemia has an ability to activate both classical pathway and lectin pathway [3] The role of IgG4 itself and complement activation pathway in IgG4-RD patients remains unsolved.

Our recent investigation revealed that elevated C5a levels in patients with active IgG4-RD inversely correlated with C3 levels and that C5a levels were low during remission (16). These data suggest that C5a is associated with the pathogenesis of IgG4-RD.

Four studies other than ours have investigated the phenotype of IgG4-RD with hypocomplementemia. These studies found specific differences in the characteristics of patients with IgG4-RD who were also diagnosed with hypocomplementemia compared with those who were not, including more frequent involvement of the lacrimal gland (11), lymph nodes (11), kidneys (4, 10, 11), pancreas (11), lungs (4, 5, 11), and prostate gland (11) and higher IgG4 serum levels (4, 11). Consistent with these results, over one-third of the patients with IgG4-RD in our study had hypocomplementemia at the initial diagnosis, and a negative correlation was observed between C3 and IgG4 levels (4). Many studies, including our results, agree that patients with IgG4-RD with hypocomplementemia have lung and kidney involvement. Further investigation is necessary to determine whether other organ involvements are associated with hypocomplementemia in IgG4-RD.

Handa et al. recently showed that sIL-2 receptor serum levels at baseline in patients with IgG4-RD significantly correlate with the number of organs involved, and soluble IL-2 receptor and IgG4 levels decrease after treatment (17). Furthermore, high sIL-2R serum levels may be a risk factor for relapse in patients with IgG4-RD (18). For the first time, we found a negative correlation between C3/C4 levels on the one hand and sIL-2R levels and the number of affected organs on the other. Serum sIL-2R may be secreted from regulatory T cells (Treg) infiltrated in affected organs because Treg cells are found around affected bile ducts and lymph nodes (17). Anti-inflammatory cytokines such as IL-10 and TGF-β are secreted by Treg (19) (20). The production of IgG4 and IgM is regulated by IL-10, and IL-10 tends to promote IgG4 production (17). In contrast, TGF-β is a major profibrotic cytokine (17).

C5a has been reported as a factor related to Treg production through C5a receptor 2 (C5aR2) (21). C5a receptors are subdivided into C5aR1 and C5aR2 (21). C5aR1 is known to have a proinflammatory role, but C5aR2 is associated with both immune-activating and immune-dampening functions (21). Human and murine C5aR2 is expressed on T cells (22). C5aR2 regulates the C5aR1-initiated signals known to inhibit Treg. A previous report showed that over expression of C5aR2 in CD4 positive T cells increased Treg/effector T cell ratio whereas C5aR2 deficiency associated with lower Treg/effector T cell ratio (22). We previously reported that serum C5a levels are elevated in patients with IgG4-RD (16). The complement system in IgG4-RD may be involved in Treg differentiation following sIL-2R production.

IgG4-RD is generally treated using corticosteroid, but the treatment other than corticosteroid has not been established. Recent report has shown that C5a receptor inhibitor is effective in ANCA-associated vasculitis (23). However, the treatment of C5a receptor inhibitors to IgG4-RD has not been reported. Considering the result of this study, C5a receptor inhibitors may be therapeutic targets in IgG4-RD patients with hypocomplementemia. Similarly, Treatments targeting IL-2 signaling may be effective to IgG4-RD. There are no drugs that directly inhibit IL-2 or IL-2R in rheumatic diseases. On the other hand, tacrolimus, which is classified as a calcineurin inhibitor, prevents activating the transcription of IL-2 gene (24). Takahashi, et al. reported the effectiveness of tacrolimus against IgG4-RD (25). Therefore, calcineurin inhibitors may be reasonable for the treatment of IgG4-RD.

**Figure 4:**
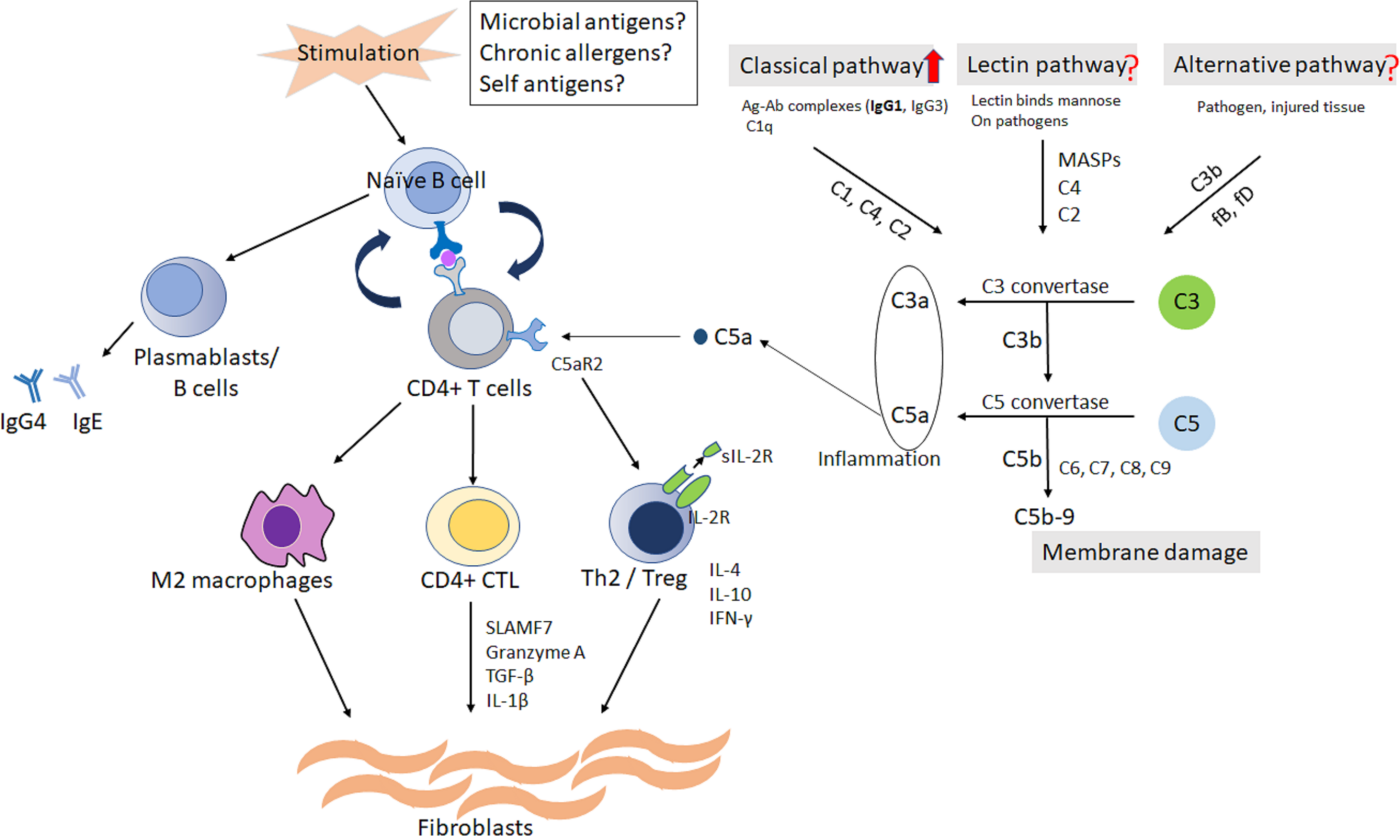
The hypothesis of pathophysiology in IgG4-RD. Naïve/memory B cells and/or dendritic cells would present antigen from possible triggers to CD4 positive T cells. The cycle of collaboration would be established between autoreactive T cells and B cells. The activated CD4 positive Th2 and regulatory T cells would produce inflammatory cytokines, resulting in activation of fibroblasts and inflammatory macrophages. Complement system, at least classical pathway, is possibly related to the differentiation to Treg from CD4 positive T cells through C5aR2. Treg may be associated with the production of sIL-2R. C, complement; CD4, cluster of differentiation 4; C5aR2; C5a receptor 2; CTL, cytotoxic T lymphocyte; Ig, immunoglobulin; IL, interleukin; IFN-γ, interferon-γ; Th2, T-helper 2; TGF-b, transforming　growth factor-b; Treg, regulatory T cell; SLAMF7, signaling lymphocytic activation molecule F7; sIL-2R, soluble interleukin-2 receptor.

Based on these considerations, the hypothesis of pathophysiology in IgG4-RD is shown in **Figure 4**. Further research is necessary to elucidate the relationship between IgG4-RD and complement system.

Our study had some limitations. First, it was a retrospective study of a relatively small number of patients, thereby the number of explanatory variables in multivariable analysis may be limited. In addition, the other complement factor including C5a could not be measured because our sample were stored at -20°C. which is unstable condition for complement factors.

Second, the proportion of patients with pancreas and kidney involvement was small; the patients had been referred from the Department of Rheumatology, thereby potentially leading to selection bias regarding the involved organs. Third, evaluating the extent of IgG4-RD only by the number of affected organs and IgG4-RD RI may be insufficient to assess disease activity because of the differentiation of radiological modality in each patient. The measurement of other biomarkers such as plasmablasts (26) and CC-chemokine ligand 18 (27) may be useful for IgG4-RD activity evaluation.

In conclusion, our study revealed that patients with IgG4-RD with hypocomplementemia had more serious disease course than those with normal complement levels. Furthermore, the strong treatments may be considered for IgG4-RD patients with hypocomplementemia.

## Data Availability Statement

The raw data supporting the conclusions of this article will be made available by the authors, without undue reservation.

## Ethics Statement

The studies involving human participants were reviewed and approved by Institutional Review Board of Nagasaki University Hospital. The patients/participants provided their written informed consent to participate in this study

## Author Contributions

YF made substantial contributions to the study concept and design with the assistance of SF. YF conducted the statistical analyses and drafted the manuscript with the assistance and supervision of KM and AK. YF, MU, ST, NI, YN, YH, TS, AO, TA, YU, AM, TO, and HW treated the patients and collected the primary data. AK critically revised the manuscript, supervised the entire study, and gave final approval to the article. All authors read and approved the final manuscript.

## Conflict of Interest

The authors declare that the research was conducted in the absence of any commercial or financial relationships that could be construed as a potential conflict of interest.

## Publisher’s Note

All claims expressed in this article are solely those of the authors and do not necessarily represent those of their affiliated organizations, or those of the publisher, the editors and the reviewers. Any product that may be evaluated in this article, or claim that may be made by its manufacturer, is not guaranteed or endorsed by the publisher.
